# NLRP3 inflammasome up-regulates major histocompatibility complex class I expression and promotes inflammatory infiltration in polymyositis

**DOI:** 10.1186/s12865-022-00515-2

**Published:** 2022-08-14

**Authors:** Ping Xia, Yu-Quan Shao, Cong-Cong Yu, Yu Xie, Zhi-Jie Zhou

**Affiliations:** 1 Department of Neurology, Sir Run Run Shaw Hospital, School of Medicine, Zhejiang University, 3 East Qingchun Road, Hangzhou, 310016 China; 2 Department of Orthopaedics, Sir Run Run Shaw Hospital, School of Medicine, Zhejiang University, 3 East Qingchun Road, Hangzhou, 310016 China; 3 Key Laboratory of Musculoskeletal System Degeneration and Regeneration Translational Research of Zhejiang Province, Hangzhou, 310016 China; 4 Medical College of Shantou University, Shantou, 515041 China

**Keywords:** NLRP3 inflammasome, Polymyositis, Major histocompatibility complex class I, MCC950, Inflammation, Autoimmune diseases

## Abstract

**Objective:**

This study was designed to investigate the role of the nucleotide-binding-domain -and leucine-rich repeat -containing (NLR) family, pyrin-domain-containing 3 (NLRP3) inflammasome in the pathogenesis of polymyositis (PM).

**Methods:**

Immunochemistry was performed to analyze the NLRP3, caspase-1 and interleukin-1 beta (IL-1β) expression in the muscle tissue of PM patients. Rat model of PM and C2C12 cell were used to investigate the potential role of NLRP3 inflammasome in PM.

**Results:**

The percentage of CD 68+ macrophages, and the expression levels of NLRP3, caspase-1 and IL-1β in the muscle tissue were elevated in 27 PM patients. LPS/ATP treatment resulted in activation of NLRP3 inflammasome and secretion of IL-1β as well as interferons (IFNs) and monocyte chemotactic protein-1 (MCP-1) in the Raw 264.7 macrophages. Meanwhile, LPS/ATP challenged activation of NLRP3 inflammasome induced overexpression of major histocompatibility complex class I (MHC-I), a key molecular of PM in the co-cultured C2C12 cells. The effect was decreased by treatment of NLRP3 inflammasome inhibitor MCC950 or siRNA of NLRP3 inflammasome. These findings suggested certain levels of IL-1β rather than IFNs up-regulated MHC-I expression in C2C12 cells. IL-1β blockade using neutralizing IL-1β monoclonal antibody or siRNA of IL-1β suppressed MHC-I overexpression. In vivo, NLRP3 inflammasome inhibition by MCC950 reduced the expression of NLRP3, IL-1β and MHC-I in the muscle tissue of PM modal rats. Also, it attenuated the intensity of muscle inflammation as well as the CRP, CK, and LDH levels in the serum.

**Conclusion:**

NLRP3/caspase-1/IL-1β axis may play an important role in the development of PM. Inhibition of NLRP3 activation may hold promise in the treatment of PM.

**Supplementary Information:**

The online version contains supplementary material available at 10.1186/s12865-022-00515-2.

## Introduction

Polymyositis (PM) is an idiopathic inflammatory myopathy characterized by symmetric proximal muscle weakness, common involvement of other organ systems such as lung and heart [1]. The accurate pathogenesis of PM has not been clearly known, yet an autoimmune basis is markedly implicated [2]. To date, the targeted auto-antigens that can initiate autoimmune process remain largely unknown and therapies for PM are broadly immunosuppressive rather than targeting specific pathogenic pathways. Some patients with PM are resistant to conventional immunosuppressive treatment generally comprising corticosteroids. Moreover, high doses of corticosteroids over a long time are associated with severe side effects [3]. Therefore, the development of safe and effective therapies for PM is essential.

In PM, the histopathology in skeletal muscle tissue implicates predominantly infiltration of inflammatory cells such as T cells and macrophages [4–6]. Another important immunological feature of PM is the up-regulation of major histocompatibility complex class I (MHC-I) [7, 8]. The CD8+ cytotoxic T cells form immunological synapses with muscle fibers that express MHC-I, resulting in myocyte necrosis [9]. Besides, MHC-I molecules themselves can potentially mediate muscle fiber damage and dysfunction even in the absence of endomysial inflammatory cells [10]. Continuous overexpression of MHC-I in muscle fibers can induce an endoplasmic reticulum stress response with accumulation of misfolded glycoproteins that may participate in muscle dysfunction [11, 12]. Therefore, abnormal expression of MHC-I in muscle fibers constitutes a major feature of PM.

The inflammatory cytokine interleukin-1β (IL-1β) is a common pro-inflammatory factor believed to be involved in PM [13]. IL-1β is mainly produced by macrophages upon activation and synthesized as an inactive precursor, which requires cleavage by active caspase-1 into its active form. And caspase-1 activity is tightly regulated by the multiprotein complexes, which are defined as inflammasomes. The nucleotide-binding-domain (NBD)-and leucine-rich repeat (LRR)-containing (NLR) family, pyrin-domain-containing 3 (NLRP3) inflammasome, found in innate immune cells, such as macrophages and dendritic cells (DCs), can activate caspase-1 through inflammasome assembly and result in the secretion of mature IL-1β [14]. It is well recognized that the activation of NLRP3 inflammasome requires two steps, priming and activation. During the first step, lipopolysaccharide (LPS), an endogenous gut-derived bacterial endotoxin, served as the first signal to induce production for precursor IL-1β and inflammasome components such as NLRP3 (15). As a second signal, adenosine triphosphate (ATP) activates an ATP-gated ion channel, P2X7 receptor (P2X7R) and promotes the inactive precursor IL-1β into mature IL-1β [15].

Recent studies have demonstrated a strong link between NLRP3/caspase-1/IL-1β axis and some autoimmune diseases including multiple sclerosis, systemic lupus erythematosus and ulcerative colitis [16–19]. However, the potential role of NLRP3/caspase-1/IL-1β axis in the development of PM is yet to be determined. If involved, it might be a new target for therapy. As it was reported that IL-1β can induce MHC-I expression in muscle cells, resulting in muscle dysfunction [12], we therefore hypothesized that NLRP3/caspase-1/IL-1β axis may promote the inflammatory development through up-regulating MHC-I expression.

Here, we have demonstrated that the NLRP3/caspase-1/IL-1β axis is active in the pathogenesis of PM. The activation of NLRP3 inflammasome in LPS/ATP stimulated Raw 264.7 macrophages induces cleavage of pro-caspase-1 into active caspase-1 and secretion of mature IL-1β, and then promotes MHC-I expression in the co-cultured C2C12 myoblasts. Genetic knockdown or pharmacological inhibition of NLRP3 inflammasome attenuates the up-regulation of MHC-I expression in C2C12 cells. Certain levels of IL-1β rather than interferons (IFNs) showed the effect of up-regulating MHC-I expression in C2C12 cells. Moreover, IL-1β blockade using neutralizing IL-1β monoclonal antibody or siRNA of IL-1β similarly alleviated MHC-I overexpression. These results indicated that NLRP3/caspase-1/IL-1β axis might play an important role in the development of PM and the inhibition of NLRP3 inflammasome might be a potential therapeutic strategy for PM. Supporting this, we have found that the specific NLRP3 inflammasome inhibitor, MCC950, alleviates the inflammatory intensity of muscle tissue in the PM model rats.

## Materials and methods

### Patients and samples

From August 2009 to October 2018, twenty-seven patients of newly diagnosed PM according to the Bohan and Peter criteria were recruited [20]. There were 9 males and 18 females, with a mean age of 43.6 ± 9.5 years (range, 25–69). Five males and 7 females without clinical or histopathological symptoms of any muscle disease were included as the control group, with a mean age of 40.3 ± 10.6 years (range, 19–61). They were diagnosed as proximal femoral fracture. And their muscle biopsies were obtained during the orthopaedic surgery, which were at least 15 cm distant from the fracture end. The clinical characteristics of all participants were recorded, including age, sex, erythrocyte sedimentation rate (ESR), C-reactive protein (CRP), creatine kinase (CK) and lactate dehydrogenase (LDH). Myositis disease activity assessment visual analogue scale (MYOACT), established by the International Myositis Assessment and Clinical Studies (IMACS) group [21], physicians’ global activity assessment on a 10 cm visual analogue scale (pVAS) [22], and evaluation of global muscle strength with manual muscle testing 8 (MMT8) [22], were used for assessing the disease activity of PM. This study was approved by the Ethic Committee of Sir Run Run Shaw Hospital, Zhejiang University and written informed consent was obtained from all participants.

Muscle biopsy specimens were obtained from the PM patients and controls after they gave written informed consent. All samples were snap-frozen in isopentane iced in liquid nitrogen immediately after surgical removal and stored at − 80 °C until analyzed.

### Animal preparation

The animal experiments were carried out according to the guidelines for the care and use of animals approved by the Ethic Committee for Animal Experiments of Sir Run Run Shaw Hospital, Zhejiang University. Forty female Sprague Dawley (SD) rats (weight 200–300 g, age 6–8 weeks) were obtained from the Laboratory Animal Center of the Sir Run Run Shaw Hospital. Rats were randomly assigned into two groups: PM model group (n = 20), and the control group (mock-induced rats, n = 20). Random numbers were generated using the standard = RAND () function in Microsoft Excel’ [23]. Animal models of PM were established as described previously [24]. Skeletal muscle harvested from New Zealand rabbit was minced and weighted. Three hundred ml of chilled 0.3 M KCl, 0.15 M sodium phosphate buffer pH 6.5 (Guba–Straub solution) was mixed with 100 g of minced muscle and kept on ice for 20 min. The homogenate was then centrifuged at 5000 rpm for 20 min at 4 °C, and the supernatant were collected. Then 15 volumes of chilled double-distilled water were added to dilute the filtrate for aggregating the myosin. After centrifugation at 5000 rpm for 20 min, the aggregated myosin was collected and dissolved in 0.5 M KCl and stored with the same volume of glycerin at -20 °C.

The SD rats were immunized by intramuscular injection of the myosin emulsified with complete Freund adjuvant (CFA) (*M. tuberculosis* 5 mg/ml). The immunogens were injected in multiple sites of the back and foot pads four times on a weekly basis. Normal saline/CFA was injected for the controls. The NLRP3 inflammasome inhibitor MCC950 (Sigma-Aldrich, St. Louis, MO, USA) dissolved in phosphate buffer saline (PBS) (n = 10) or the PBS alone (n = 10) was randomly administered to the rats intraperitoneally on the day after last injection of immunogens and then daily at a dose of 10 mg/kg of body weight for 7 days. The injection of MCC950 (n = 10) or PBS (n = 10) was also applied to the control group. Rats were anesthetized with pentobarbital sodium (40 mg/kg, intraperitoneal injection) and sacrificed one week after the last injection. For each rat, muscle and blood samples obtained were for analysis. Histologic severity of inflammation, and protein expression level of NLRP3, IL-1β, and MHC-I in the muscle tissue, as well as serum level of CRP, CK and LDH were evaluated**.**

### Histological analysis

Myositis was defined as mononuclear cell infiltration spreading around muscle fibers, including at least 1 necrotic muscle fiber [25]. Muscle section samples were fixed on 7 μm serial cryostat sections and stained with hematoxylin–eosin (H&E) in accordance with the standard protocol. The histologic severity of inflammation of each rat muscle block was evaluated by a semi-quantitative scoring system as described previously [24, 25]. Grade 1: < 5 muscle fibers involved; grade 2: a lesion involving 5–30 muscle fibers; grade 3: a lesion involving a muscle fasciculus; grade 4: diffuse extensive lesions. When multiple lesions with the same grade were found in a single muscle block, 0.5 point was added to the grade. A mean score of bilateral quadriceps was calculated and used for as the score for each rat.

Two independent observers blinded to the experimental protocols evaluated all muscle sections. Disagreements were resolved by discussion.

### Immunohistochemical staining and immunofluorescence

Immunohistochemical staining and immunofluorescence were performed on 7 μm serial cryostat sections from the muscle samples and dipped in acetone for 10 min at room temperature. For immunohistochemical staining, sections were blocked with 10% goat serum albumin in PBS for 15 min at room temperature and incubated with primary NLRP3 antibody (1:200, Santa Cruz Biotechnology Cat# sc-66846, RRID: AB_2152446), caspase-1 antibody (1:200, AbcamCat# ab179515, RRID: AB_2884954), IL-1β antibody (1:200, Santa Cruz Biotechnology Cat# sc-7884, RRID: AB_2124476), CD 68 antibody (1:200, AbcamCat# ab125212, RRID: AB_10975465), interferon-alpha (IFN-α) antibody (1:100, Abcam, ab193055), interferon-beta (IFN-β) antibody (1:100, Abcam, ab140211), or interferon-gamma (IFN-γ) antibody (1:100, Abcam, ab25101), overnight at room temperature. After incubation, the samples were washed in PBS. Then the second antibody (goat anti-rabbit IgG, CWBio Cat# CW0103, RRID: AB_2814709) labeled with horseradish peroxidase was added for 30 min at − 80 °C. After that, the samples were washed in PBS again. The second antibody was visualized using 3,3,-diaminobenzidine tetrahydrochloride (DAB kit, Wuxi Leyuan, China) as a chromogenic substrate solution. Sections were counterstained with hematoxylin, and mounted in gelatine. All immunohistochemical stains for PM patients and controls were analyzed on coded slides. They were evaluated in the entire area of two cross-sections from one biopsy under 400 × magnification. Two neuropathologists blinded to the clinical data evaluated each biopsy and counted numbers of total inflammatory cells and CD68, NLRP3, caspase-1 or IL-1β positive cells.

For immunofluorescence labeling, the fluorescein isothiocyanate (FITC)-labeled anti-human HLA-ABC antibody (clone W6/32, Serotec, Oxford, UK) was used. After blocked with 10% goat serum albumin in PBS at room temperature for 15 min, the sections were incubated with 1:50 dilution of the anti-HLA antibody at room temperature. The grade of MHC-I positive staining was assessed using the criteria recommended by van der Pas and colleagues [7]: grade−, undetectable in myofibers, but present on capillaries; grade+, both capillaries and myofiber sarcolemma are stained, but the capillaries can still be identified easily; and grade++, both capillaries and myofiber sarcolemma are stained, but capillaries can no longer be identified.

### Cell culture and Small interference RNA (siRNA) knockdown

The Raw 264.7 mouse macrophages used in this study were stable ectopically expressed ASC and cultured in DMEM containing 10% fetal calf serum (FCS), supplemented with 200 U/ml penicillin and 100 μg/ml streptomycin, at 37 °C in 5% CO2. siRNA targeting NLRP3 and IL-1β were designed and constructed by GenePharama Corporation (Shanghai, China). Raw 264.7 macrophages were transfected with siRNA targeting NLRP3 or IL-1β using Lipofectamin3000 reagent (Invitrogen, CA) and OPTI-MEMI (Invitrogen). Transfected cells were incubated with differentiated C2C12 cells for 48 h.

### Indirect co-culture assay

For indirect co-culture assay, C2C12 mouse myoblasts (1 × 10^5^ cells), were seeded onto the lower compartment of 6-well transwell plates (0.4 μm pore size, Corning, NY) and incubated until approximately 70–80% confluence with DMEM containing 10% fetal calf serum (FCS), supplemented with 200 U/ml penicillin and 100 μg/ml streptomycin, at 37 °C in 5% CO_2_. Then the cells were differentiated in DMEM containing 2% horse serum for about 48 h. Raw 264.7 mouse macrophages (1 × 10^5^ cells) were seeded onto the upper insert of 6-well transwell plate for 24 h. Then the C2C12 cells were co-cultured with the Raw 264.7 macrophages in DMEM containing 10% FCS at 37 °C in a humidified atmosphere with 5% CO_2_.In treatment groups, the Raw 264.7 macrophages were pretreated with NLRP3-siRNA, MCC950 (Sigma-Aldrich, 10 μM), or IL-1β-siRNA. Or in the co-culture system, the C2C12 cells were treated with additional neutralizing monoclonal antibody of IL-1β (1 μg/ml, clone 7E3, InvivoGen).

### ELISA

Serum, cell supernatant or muscle homogenate levels of IL-1β, tumor necrosis factor alpha (TNF-α), IFN-α, IFN-β, IFN-γ,and monocyte chemotactic protein-1 (MCP-1), were measured using the ELISA kits (Elabscience), according to the manufacturer’s instructions.

### RNA isolation and quantitative real-time polymerase chain reaction (qRT-PCR)

Total RNA was isolated from C2C12 cells using TRIZOL (Invitrogen). cDNA was synthesized using 4 μg of RNA from each sample, 4 μl of 5 × PrimeScript RT Master Mix and 3 μl of RNase-free distilled H_2_O in a total volume of 20 μl. Reverse transcription polymerase chain reaction was performed using SYBR Green qPCR Master Mix (Takara Bio, Otsu, Japan) with a LightCycler (CFXTM Touch; Bio-Rad, Hercules, CA, USA). The resulting cDNA product was stored at -20 °C until use. The total volume (10 μl) of each PCR reaction contained 10 μl Mix, 2.5 μl ddH_2_O, 2 μl cDNA and 10 μM of each of the forward and reverse primers. The PCR conditions consisted of denaturation at 95 °C for 2 min, followed by 95 °C for 10 s, and 60 °C for 30 s for 40 cycles. The primer sequences used are listed in Additional file 1: Table S1.

### Western blot analysis

Cells were lysed and total proteins were quantified. Samples (30–50 μg) were separated by 10% SDS-PAGE (stacking gel 50 V, separating gel 100 V) and transferred to a polyvinylidene fluoride membrane (100 V for 75 min), and then blocked in 5% defatted milk for 2 h at room temperature. After incubation with primary antibodies of rabbit anti-NLRP3 (1:500, Santa Cruz Biotechnology Cat# sc-66846, RRID: AB_2152446), anti-caspase-1 (1:500, AbcamCat# ab179515, RRID: AB_2884954), anti-IL-1β (1:500, Santa Cruz Biotechnology Cat# sc-7884, RRID: AB_2124476), and anti-MHC-I (1:500; Abcam Cat# ab52749, RRID: AB_2042338) overnight at 4 °C, they were incubated in a goat anti-rabbit horse radish peroxidase (HRP)-conjugated secondary antibody (1:2000; CWBio Cat# CW0103, RRID: AB_2814709) for 1 h. The protein bands were assayed by chemiluminescence (BeyoECL; Beyotime, Zhengzhou, China). Densitometry analysis of protein levels was performed using Gel-pro Image Analysis software (Wayne Rasband, National Institutes of Health, Bethesda, MD, USA).

### Statistical analysis

All statistical analysis was performed using SPSS 18.0 software (Illinois, USA). The data were presented as mean ± standard deviation (SD). Double-tailed Student’s *t*-test or one-way analysis of variance (ANOVA) was used to assess the differences between groups Spearman rank correlation coefficient was used for correlation analysis. *P* < 0.05 was considered statistically significant.

## Results

### The level of inflammation is increased in the PM patients and the NLRP3/caspase-1/IL-1β axis is active in their muscle samples

**Fig. 1:**
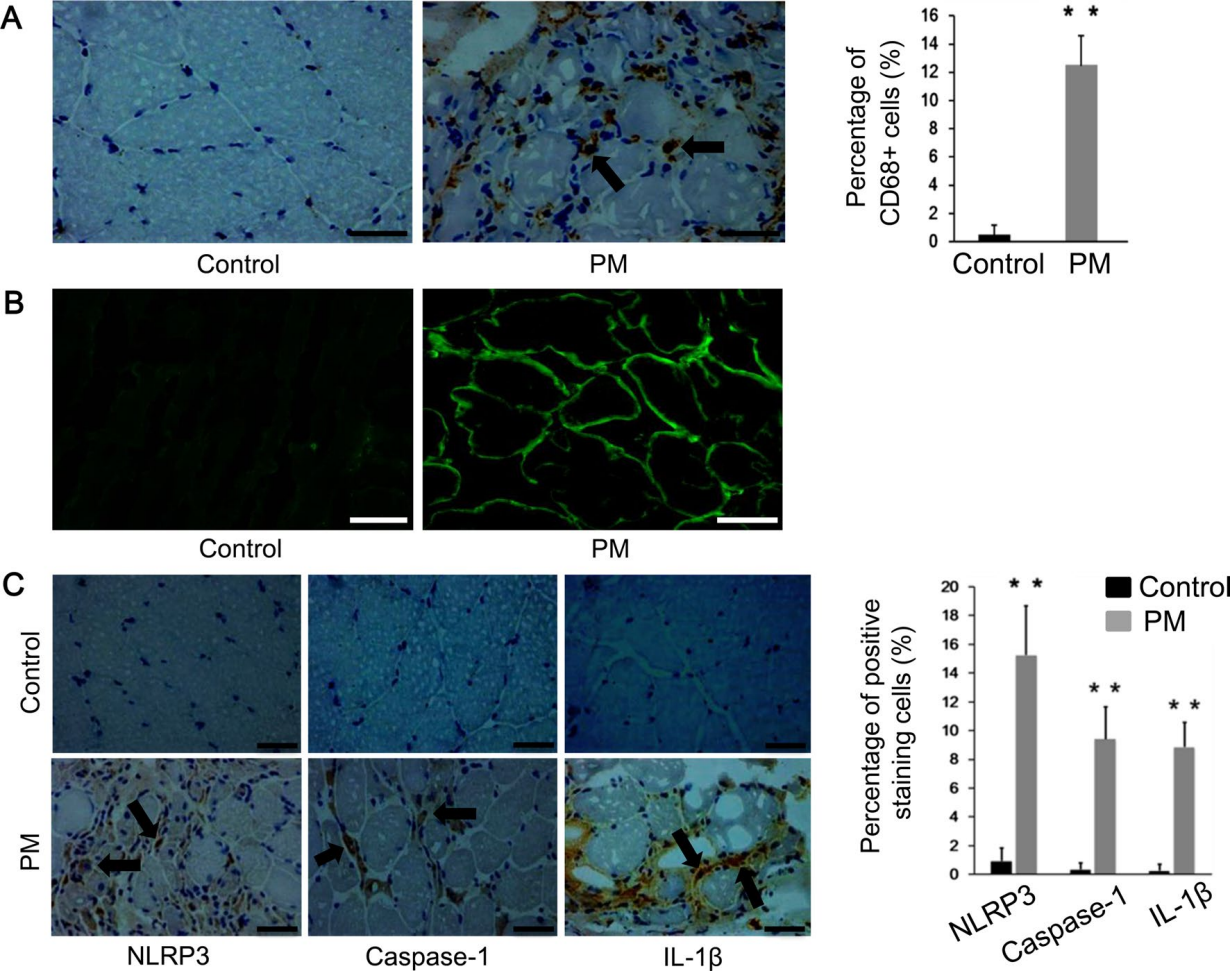
The level of inflammation is increased in the PM patients and the NLRP3/caspase-1/ IL-1β axis is active in their muscle samples. **A** Immunohistochemistry analysis showed higher percentage of CD68+ cells (solid black arrows) in the muscle biopsies in the PM patients than in the controls. **B** Immunofluorescence staining showed positive MHC-I staining (grade+ ~ ++) in the PM patients, and negative in the controls (grade−). **C** Immunohistochemistry analysis of the muscle biopsies showed higher expression of NLRP3, caspase-1 and IL-1β (solid black arrows) in the PM patients than in the controls. **p < 0.001; scale bars, 50 μm (original magnification, ×400)

Demographic and disease characteristics of the PM patients and controls are shown in Additional file 1: Table S2. There were no significant differences in sex and age between them. However, the ESR, CRP, CK and LDH values in the serum were significantly higher in the PM patients. Then the muscle biopsy samples of all subjects in the two groups were analyzed. There was significantly higher percentage of macrophages among the infiltrated inflammatory cells, identified as CD68+ cells, in the PM patients than in the controls (Fig. 1A). The presence of positive staining for MHC-I was detected in muscle fibers of PM patients (grade+  ~ ++), but not in the controls (grade−) (Fig. 1B). PM group showed statistically higher expression of NLRP3, caspase-1, and IL-1β (positive stained infiltrating cells to the total infiltrating cells), and the majority of stained cells were the macrophages (Fig. 1C). No positive staining for IFN-α, IFN-β or IFN-γ was observed in the muscle samples of both PM and control groups (Additional file 1: Fig. S1). These results indicated the possible involvement of the NLRP3/caspase-1/IL-1β axis in the pathogenesis of PM.

### NLRP3 inflammasome is activated in LPS/ATP stimulated Raw 264.7 macrophages, and NLRP3 inflammasome activation can induce secretion of IL-1β as well as IFNs and MCP-1

**Fig. 2:**
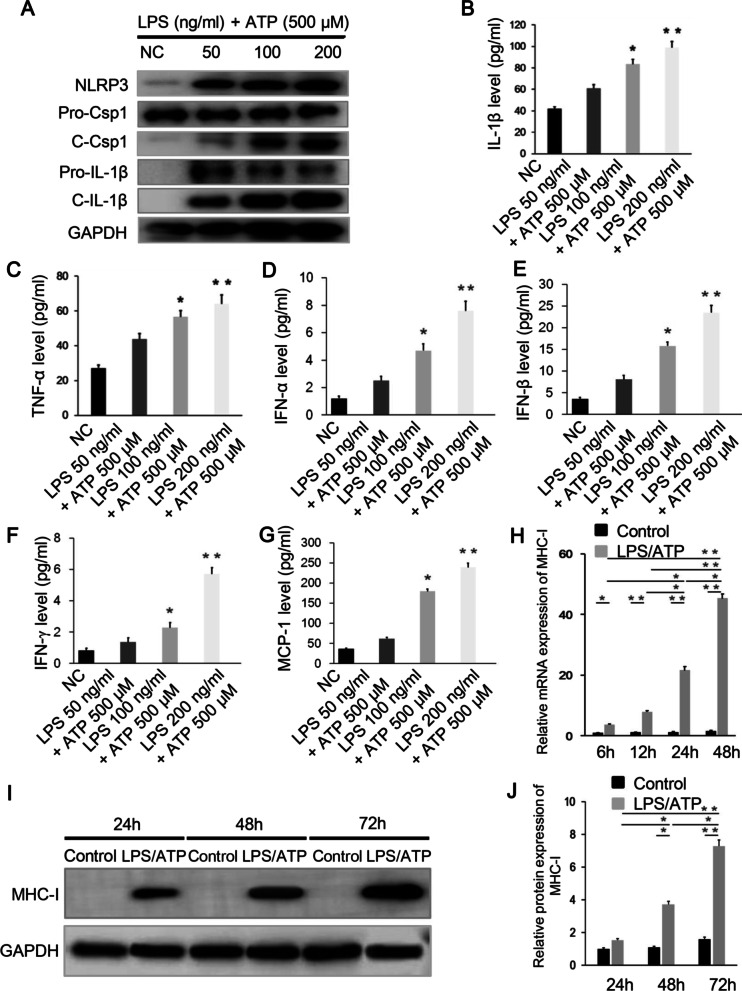
LPS/ATP stimulation activates NLRP3/caspase-1/IL-1β axis and promotes MHC-I expression. **A** Western blot analysis showed increased expression of NLRP3, pro-caspase-1, cleaved caspase-1, pro-IL-1β and mature IL-1β in the Raw 264.7 macrophages under LPS/ATP stimulation. **B**, **C** ELISA analysis showed that the expression of IL-1β and TNF-α in the Raw 264.7 macrophages were up-regulated by LPS/ATP. **D**–**G** ELISA analysis showed elevated levels of IFNs and MCP-1 produced by the stimulated macrophages. **H** The mRNA expression of MHC-I in the co-cultured C2C12 cells was increased under LPS/ATP stimulation and peaked at 48 h. **I**, **J** The protein expression of MHC-I in the co-cultured C2C12 cells was increased under LPS/ATP stimulation and peaked at 72 h. *p < 0.01, **p < 0.001, compared with control group or compared between the groups connecting with the line

To analyze the role of NLRP3/caspase-1/IL-1β axis in PM, we treated the Raw 264.7 macrophages with LPS and ATP in 6-well plates. We found that the protein expression of NLRP3 and caspase-1 (p10) were increased in response to LPS for 6 h and ATP for another 1 h co-treatment in the Raw 264.7 macrophages, as shown by western blot analysis. The effect of co-treatment peaked at 24 h, with 200 ng/ml LPS and 500 μM ATP (Fig. 2A). IL-1β is a major pro-inflammatory product of NLRP3 inflammasome. To investigate the cytokine-releasing activity of NLRP3 inflammasome, we next detected IL-1β levels in the culture supernatant at different intervention conditions by ELISA. It revealed that the IL-1β levels increased upon LPS/ATP treatment, and peaked when treated with 200 ng/ml LPS and 500 μM ATP (Fig. 2B). A similar trend of TNF-α expression was found under the stimulation, and the expression level was lower than that of IL-1β (Fig. 2C). LPS/ATP treatment also induced relatively low level of IFN-α, IFN-β, and IFN-γ (Fig. 2D–F), in comparison to a high level of MCP-1 (Fig. 2G). Together, these results revealed that LPS/ATP stimulation could activate NLRP3 inflammasome and subsequent pro-caspase-1 cleavage and IL-1β secretion in the Raw 264.7 macrophages. Also, LPS/ATP stimulation on the Raw 264.7 macrophages produced elevated levels of IFNs and MCP-1.

### LPS/ATP treated Raw 264.7 macrophages promote MHC-I expression in C2C12 cells

MHC-I overexpression in sarcolemma of skeletal muscle fibers is considered as a remarkable feature of PM and a key molecular mechanism to develop PM. We hypothesized that NLRP3 inflammasome activation may induce MHC-I overexpression in C2C12 cells. To further evaluate the assumption, we assessed the MHC-I expression in C2C12 cells after indirectly co-cultured with LPS/ATP stimulated Raw 264.7 macrophages. The mRNA expression of MHC-I in C2C12 cells was significantly enhanced after the Raw 264.7 macrophages challenged with 200 ng/ml LPS and 500 μM ATP, and peaked at 48 h (Fig. 2H). The protein expression of MHC-I was correspondingly promoted and peaked at 72 h treatment (Fig. 2I, J).

### Down-regulation of NLRP3 attenuates MHC-I expression in C2C12 cells

**Fig. 3:**
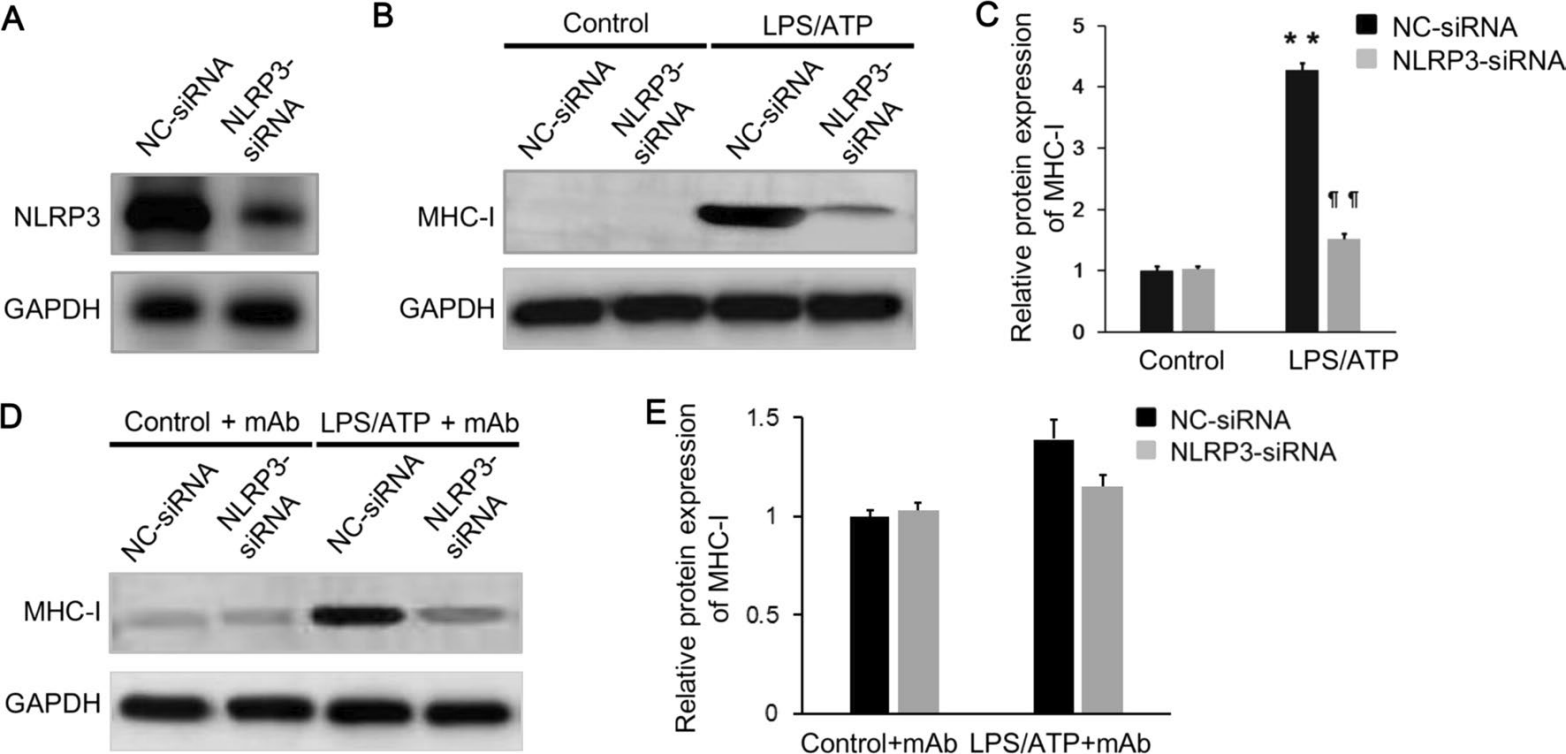
Genetic knockdown of NLRP3 suppresses MHC-I overexpression in C2C12 cells. **A** Raw 264.7 macrophages were transfected with siRNA targeting NLRP3, and the transfection efficiency was measured by western blot analysis. **B**, **C** Raw 264.7 macrophages pretreated with NLRP3-siRNA were stimulated with 200 ng/ml LPS + 500 μM ATP, and then the expression of MHC-I in the co-cultured C2C12 cells was significantly reduced compared with those pretreated with NC-siRNA, as revealed by western blot. **D**, **E** The stimulated Raw 264.7 macrophages were pretreated with NLRP3-siRNA or NC-siRNA, and the co-cultured C2C12 cells were added with neutralizing IL-1β monoclonal antibody. No differences in the expression of MHC-I in the co-cultured C2C12 cells were found between those groups stimulated with 200 ng/ml LPS + 500 μM ATP or with PBS, as showed by western blot. **p < 0.001, compared with control group; ¶¶*p* < 0.001, compared with before treatment

To investigate the role of NLRP3 inflammasome on inducing MHC-I expression in C2C12 cells, we co-cultured C2C12 cells with Raw 264.7 macrophages. The Raw 264.7 macrophages were stimulated with 200 ng/ml LPS and 500 μM ATP. siRNA was used for depleting the expression of NLRP3 in the Raw 264.7 macrophages (Fig. 3A). Western blot analysis revealed that NLRP3 gene expression knockdown significantly reduced MHC-I up-regulation (Fig. 3B, C). Further, IL-1β blockade for treating the C2C12 cells using neutralizing IL-1β monoclonal antibody (mAb) could similarly inhibited its MHC-I up-regulation (Fig. 3D, E). These results supported our hypothesis that NLRP3/caspase-1/IL-1β axis might be involved in mediating MHC-I expression in C2C12 cells.

### Pharmacological inhibition of NLRP3 inflammasome alleviates MHC-I overexpression in C2C12 cells

**Fig. 4:**
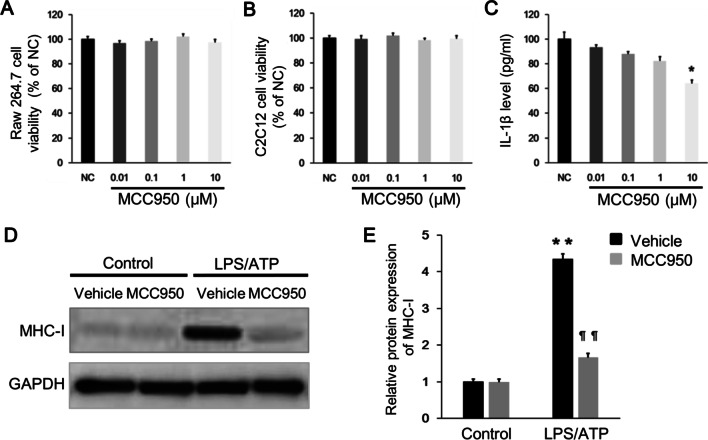
Pharmacological inhibition of NLRP3 inflammasome using MCC950 effectively suppresses MHC-I overexpression in C2C12 cells. **A**, **B** Cell viability assay revealed no significant cytotoxicity of MCC950 to the Raw 264.7 cells and C2C12 cells, at the dosage from 0.01 to 10 μM. **C** LPS/ATP stimulated Raw 264.7 macrophages were treated by MCC950 with dosage from 0.01 to 10 μM, and the IL-1β level in the cell supernatant was significantly decreased at the dosage of 10 μM. **D**, **E** LPS/ATP stimulated Raw 264.7 macrophages were pretreated by 10 μM MCC950, and the MHC-I expression in the co-cultured C2C12 cells was significantly suppressed as showed by western blot. *p < 0.01, **p < 0.001, compared with control group; ¶¶*p* < 0.001, compared with before treatment

We co-cultured Raw 264.7 macrophages and C2C12 cells for 72 h. MCC950, a selective NLRP3 inflammasome inhibitor, was used to block the activation of NLRP3 inflammasome in Raw 264.7 macrophages. Raw 264.7 macrophages were first primed with LPS for 6 h then pre-treated with MCC 950 for 30 min and lastly stimulated with ATP for another 1 h. Cell viability assay (as CCK8) showed that MCC950 did not induce significant cytotoxicity in Raw 264.7 cells and C2C12 cells at the dosage from 0.01 μM to 10 μM (Fig. 4A, B). Moreover, 10 μM MCC950 significantly decreased IL-1β level in the cell supernatant of LPS/ATP stimulated Raw 264.7 macrophages (Fig. 4C). Western blot analysis indicated that 10 μM MCC950 effectively alleviated LPS/ATP induced MHC-I up-regulation in the co-cultured C2C12 cells (Fig. 4D, E). These results suggested that pharmacological inhibition of NLRP3 inflammasome could inhibit MHC-I overexpression in C2C12 cells.

### IL-1β is crucial for MHC-I up-regulation in C2C12 cells

**Fig. 5:**
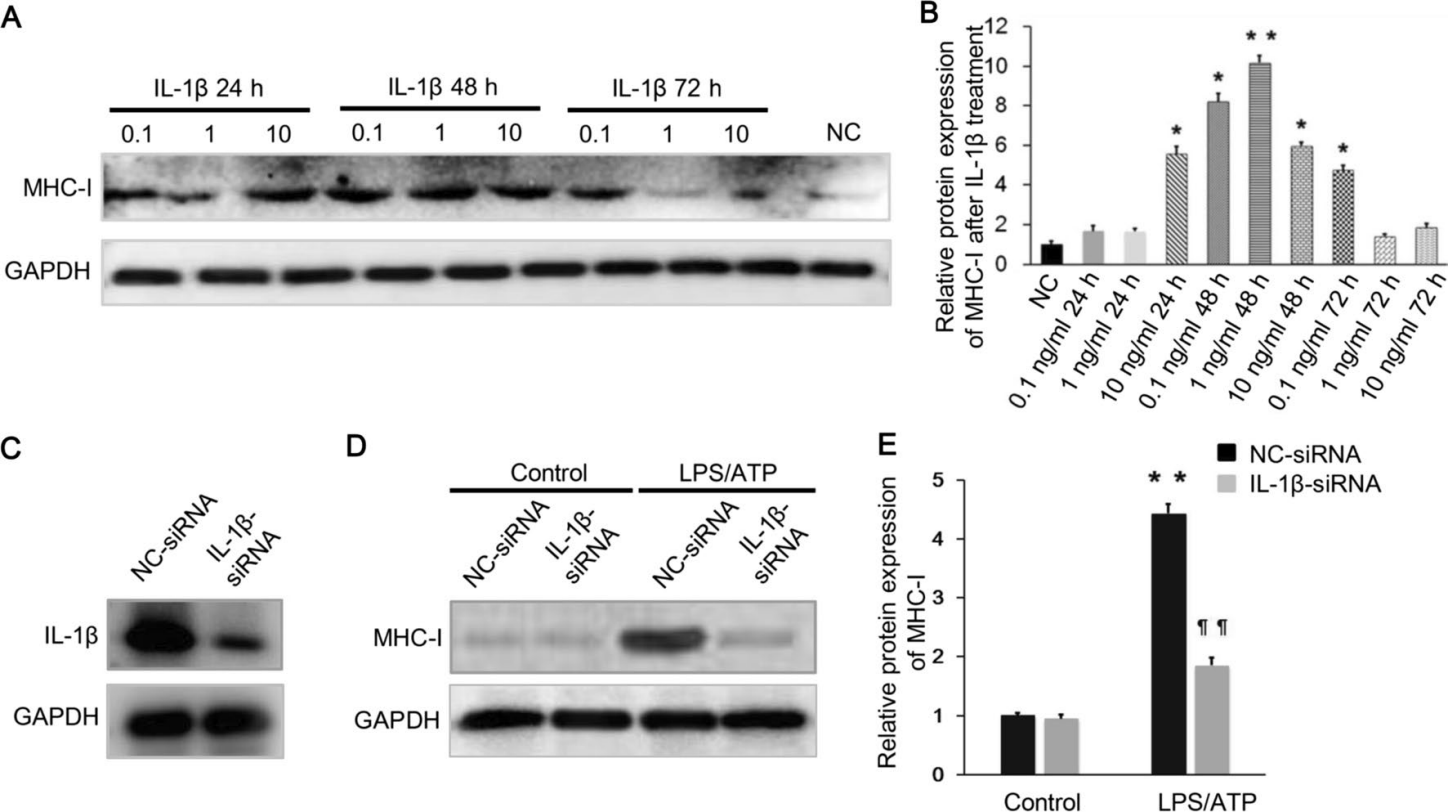
IL-1β is crucial for MHC-I up-regulation in C2C12 cells. **A**, **B** Recombinant IL-1β was added into the C2C12 cells. The expression of MHC-I in C2C12 cells peaked when treated by 1 ng/ml IL-1β for 48 h (n = 5). **C** Raw 264.7 macrophages were transfected with siRNA targeting IL-1β, and the transfection efficiency was measured by western blot analysis. **D**, **E** LPS/ATP stimulated Raw 264.7 macrophages were pretreated with IL-1β-siRNA or NC-siRNA, and the MHC-I expression in the co-cultured C2C12 cells was significantly lower with the former than with the latter pretreatment, as analyzed by western blot. *p < 0.01, **p < 0.001, compared with control group; ¶¶*p* < 0.001, compared with before treatment

To explore the exact correlation between NLRP3 inflammasome activation and MHC-I overexpression, we further examined the functional significance of IL-1β on MHC-I overexpression of C2C12 cells by adding recombinant IL-1β into C2C12 cells. The expression level of MHC-I protein was significantly enhanced when the C2C12 cells were challenged with pro-inflammatory cytokine IL-1β. The effect was most obvious when the C2C12 cells were treated with 1 ng/ml IL-1β for 48 h (Fig. 5A, B). However, only weak effects on upregulating MHC-I expression in C2C12 cells were observed for the treatment using IFN-α (80 pg/ml), IFN-β (200 pg/ml), or IFN-γ (60 pg/ml) for 48 h (Additional file 1: Fig. S2). While using specific siRNA to knock down the IL-1β gene of Raw 264.7 macrophages (Fig. 5C), we found that the protein level of MHC-I in C2C12 cells was significantly decreased (Fig. 5D, E), suggesting a crucial role of IL-1β in the inflammatory process.

### NLRP3 inflammasome inhibition using MCC950 attenuates the intensity of inflammation and MHC-I expression in PM model rats

**Fig. 6:**
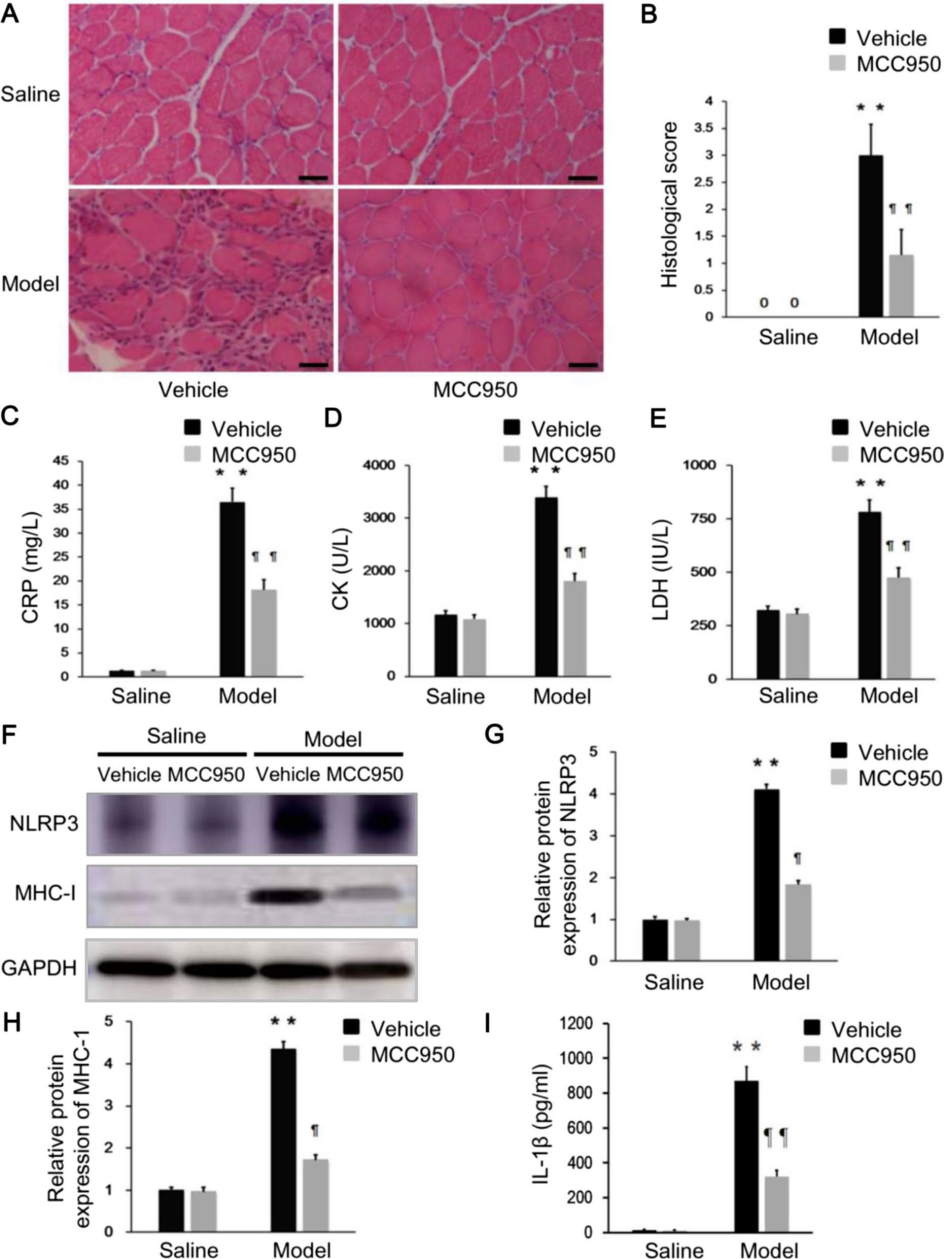
MCC950 alleviates the intensity of inflammation and MHC-I expression in PM model rats. **A**, **B** The muscle section samples of PM modal rats showed a substantial reduction in muscle histological inflammation scores after treated by MCC950. **C**–**E** Serum CRP, CK, and LDH levels were significantly reduced after MCC950 treatment in PM modal rats. **F**–**I** The expression of NLRP3, MHC-I, and IL-1β were effectively reduced in the muscle tissue of PM model rats by MCC950 treatment. **p < 0.001, compared with control group; ¶*p* < 0.01, ¶¶*p* < 0.001, compared with before treatment. Scale bars, 50 μm (original magnification, ×400)

In light of our study suggesting that the NLRP3/caspase-1/IL-1β axis is correlated with inflammatory severity in PM patients and the MHC-I expression is NLRP3/caspase-1/IL-1β axis-dependent in vitro, we assessed the ability of MCC950 to prevent NLRP3 inflammasome activation and to alter the inflammatory response in PM model rats. PM model rats treated with MCC950 displayed a substantial reduction in muscle histological inflammation scores, compared with PM model rats treated with PBS (Fig. 6A, B). We also found that MCC950 was effective in reducing serum CRP, CK, and LDH levels in PM model rats (Fig. 6C–E). Furthermore, the NLRP3 and MHC-I protein levels in muscle tissues were significantly reduced after MCC950 administration (Fig. 6F–H), as well as the IL-1β level (Fig. 6I). These suggested that MCC950 might regulate muscle inflammation via suppressing NLRP3/caspase-1/IL-1β axis in PM modal rats.

## Discussion

Given our limited understanding of PM etiology and the absence of targeted therapies, it is highly important to understand the mechanisms of the disease. In the present study, we investigated the role of NLRP3 inflammasome in PM patients and showed that in a cohort of our patients diagnosed of newly onset PM, the percentage of CD68+ cells, and the expression of NLRP3, along with caspase-1 and IL-1β were elevated. In vitro, we showed that LPS/ATP treatment resulted in the activation of NLRP3 inflammasome and secretion of IL-1β as well as IFNs and MCP-1 in the Raw 264.7 macrophages. Meanwhile, LPS/ATP challenged activation of NLRP3 inflammasome induced MHC-I overexpression in C2C12 cells. Genetic knockdown or pharmacological inhibition of NLRP3 inflammasome could alleviate MHC-I up-regulation in C2C12 cells. Certain levels of IL-1β rather than IFNs showed the effect of up-regulating MHC-I expression in C2C12 cells. Further, IL-1β blockade using neutralizing IL-1β monoclonal antibody or siRNA of IL-1β suppressed MHC-I overexpression. In vivo, the specific NLRP3 inhibitor, MCC950, also effectively attenuated the MHC-I expression and inflammatory response in PM model rats.

The overexpression of MHC-I is an early event in many autoimmune diseases, particularly in tissues such as muscles, neuronal cells and pancreatic β cells that show little or no constitutive expression [12]. Muscle fibers in normal individuals do not express detectable levels of MHC-I antigens. However, muscle fibers in autoimmune myositis intensively overexpressed MHC-I, particularly in PM [26]. Therefore MHC-I is considered as an effective molecular marker for PM. The increased level of MHC-I molecule in the skeletal muscle is associated with muscle fiber damage and/or induction of endoplasmic reticulum stress, contributing to muscle dysfunction [27, 28]. Moreover, a previous study showed that the overexpression of MHC-I alone in the mice skeletal muscle could lead to a self-sustaining inflammatory process involving muscle fiber damage and infiltration of mononuclear cells with the most of the characteristic features of human PM, and suggested that the up-regulation of MHC-I may initiate a cascade of events typical of muscle inflammation in the spontaneous human disease [29]. So far, the regulatory mechanism of MHC-I expression in PM remains unclear.

NLRP3 inflammasome, which is activated upon signs of cellular “danger” to trigger innate immune defense through the maturation of pro-inflammatory cytokines such as IL-1β and IL-18, is reported to be closely associated with autoimmune diseases [16–19]. The function of NLRP3 inflammasome and its significant role have been demonstrated in several autoimmune diseases, including rheumatoid arthritis [30, 31], ankylosing spondylitis [32, 33], and multiple sclerosis [17]. Therefore, NLRP3 may be a promising therapeutic target for autoimmune diseases. Nevertheless, investigations regarding the potential role of NLRP3 inflammasome in PM are largely lacking. Only one study recently revealed that NLRP3 inflammasome may be implicated in the pathogenesis of PM, supported by the elevated expression level of NLRP3, IL-1β and IL-18 in a series of PM patients [34]. Our results confirmed that the NLRP3/caspase-1/IL-1β axis was active in the PM patients.

We then showed that LPS/ATP induced NLRP3 inflammasome activation was in line with MHC-I expression in C2C12 cells. Knockdown of NLRP3 gene by siRNA or pharmacologically suppressing the activation of NLRP3 inflammasome using MCC950 could down-regulate MHC-I expression. These results suggested that NLRP3 inflammasome might be involved in regulating MHC-I expression under inflammatory stimulus.

IL-1β, the secretion of which is tightly controlled by inflammasomes that aggregate upon exposure to specific activators, is believed to be an important pro-inflammatory factor contributing to the pathogenesis of PM. The level of IL-1β was higher in PM patients than that in controls [13, 35], as was shown in this study; and was significantly decreased after steroid therapy [36]. In PM murine models, the IL-1β expression in the muscle tissues increased as the severity of myositis peaked and the inflammatory intensity could be relieved by IL-1 antagonism [25]. More importantly, pro-inflammatory cytokines including IL-1α, IL-1β and TNF-α can induce MHC-I expression in normal human skeletal muscle cells [37]. We also found that IL-1β up-regulated MHC-I expression in the muscle cells. Further, genetically inhibiting the expression of IL-1β or IL-1β blockade using neutralizing IL-1β monoclonal antibody could attenuate the up-regulation of MHC-I in C2C12 cells. Together, these indicated that NLRP3 inflammasome might regulate MHC-I expression through the NLRP3/caspase-1/IL-1β axis.

The active involvement of IL-1 in the inflammatory process leads to the development of targeted therapies using IL-1 blockade. Yet the clinical results of IL-1β blockade were mixed [38–40]. Zong et al. [38] reported a satisfactory outcome of anakinra treatment in patients with refractory inflammatory myopathies. In their study, 3 out of 6 PM patients, 3 out of 4 dermatomyositis (DM), and 1 out of 5 inclusion body myositis (IBM), showed clinical response to anakinra treatment. However, other reports using IL-1β blockade (canakinumab or anakinra) to treat IBM showed less satisfactory results [39, 40]. Further understanding of the molecular mechanisms of IL-1β in the pathogenesis of myositis is required.

Beyond IL-1β, recently studies suggest several cytokines and chemokines as important key molecules in the pathogenic mechanisms of inflammatory myopathies, such as IFNs and MCPs [41, 42]. In particular, IFNs are reported as potent inducer of MHC-I in myositis [43]. We therefore investigated the expression of IFN-α, IFN-β, IFN-γ, and MCP-1 in the LPS/ATP stimulated macrophages. Elevated expression levels of IFNs and MCP-1 were detected, but the levels of IFNs were much lower than that of IL-1β (Fig. 2). Our result was in agreement with other findings that IFNs were less abundantly produced by the stimulated macrophages, compared to IL-1β or TNF-α [44–46]. However, the degree of increasing in the expression level of IFNs was obviously higher than that of IL-1β in PM patients [47–49]. The inconsistency in the expression of IFNs as compared with IL-1β between cells experiments and in vivo conditions may be interpreted as follows. IFN-γ is produced almost exclusively by natural killer (NK) cells and certain subpopulation of activated T cells [41, 42]. And the major source of IFN-α most probably is the plasmacytoid dendritic cells that are present in all inflammatory myopathies, especially in DM [41]. Therefore, since the macrophages are not the main resource of IFNs [50], it is reasonable that the expression of IFNs produced by macrophages was not as high as it was in PM. Supporting that, Rostasy et al. [50] found IFN-γ was robustly expressed in inflammatory cells located primarily in the endomysium in PM, but the macrophages in the vicinity of injured muscle fibers were devoid of IFN-γ. A previous study reported that IFN-γ appears to have higher efficacy than other cytokines in up-regulating MHC-I expression in muscle cells [37]. However, in that study, the concentration of IFN-γ used to stimulate muscle cells was much higher than that of IL-1β, TNF-α or MCP-1 [37]. When the concentrations of IFNs and IL-1β were selected according to their levels produced by our stimulated macrophages, the IFNs showed much weaker effects on up-regulating MHC-I expression, compared with IL-1β (Additional file 1: Fig. S2).

What’s more, conflicting data exist regarding the IFNs expression in inflammatory myopathies. Some investigations using immunoassay analysis only detected IFNs expression in a minority of the muscle sample in patients with myositis [48, 50, 51]. We also did not detect the expression of IFN-α, IFN-β, or IFN-γ in our PM muscle samples (Additional file 1: Fig. S1). So, although IFNs are expressed in inflammatory cells in patients with inflammatory myopathies, they are only detected in occasional patients and only in a few cells, rendering IFNs less likely as potent candidates in the pathogenic mechanisms of myositis disorders [42, 52].

Similarly for chemokines, although they govern the migration of leukocytes from the blood to tissue inflammation sites, and play a critical role in promoting muscle inflammation and tissue damage in inflammatory myopathies [41], their individual contribution to the inflammatory process is undefined. In particular, MCP-1 of a much higher level (100 ng/ml) than ours showed little effect on up-regulating MHC-I expression [37]. Therefore, in our co-culture system, MCP-1, as well as IFNs, were supposed to have a minor role in enhancing MHC-I expression. However, this is not necessarily applicable to conditions in vivo. To further study whether inhibiting NLRP3 inflammasome could suppress inflammation in PM, we used a specific NLPR3 inhibitor, MCC950, in PM model rats. The PM model rats expressed high level of NRLP3, IL-1β and MHC-I in the muscle tissue, corroborating observations in our PM patients. We observed that NLRP3 blockade not only effectively down-regulated the downstream IL-1β secretion and the protein level of MHC-I, but also resulted in a marked reduction in histologic severity of muscle inflammation as well as in serum level of CRP, CK and LDH. Of note, MHC-I is a key molecular marker for PM that was involved in inflammation processes and muscle dysfunction. These findings pointed towards an immunomodulatory function of NLRP3/caspase-1/IL-1β axis likely through regulating MHC-I production, and consequently aggravating the inflammatory intensity of the muscle tissue.

There were some limitations in our study. First, it is uncertain that the macrophages recruitment and inflammasome activation in patients must be prior to MHC-I up-regulation. A variety of cytokines and chemokines produced by the inflammatory cells in PM might induce MHC-I up-regulation before the macrophages were activated. MHC-I over-expression in muscle cells was also observed in the absence of inflammatory infiltrates in adult inflammatory myopathies, both in early disease an in late inactive disease [53]. Second, the clinical relevance of our co-culture experiments was inferior to that using the primary myoblasts and monocyte/macrophages from the PM and control patients. However, when the patient samples are not available, cell experiments using Raw 264.7 macrophages and C2C12 cells are the one of the most common strategies for studying inflammatory myopathies [54–56]. Third, our finding that IFNs contributed less to the up-regulation of MHC-I was limited to cell experiments, and could not be directly extended to conditions in vivo. And we did not investigate whether the production of IFNs was directly by NLRP3 inflammasome or via IL-1β in an autocrine way. Nevertheless, to some extent, this finding indicated that the involvement of macrophages in the pathogenesis of PM might be via NLRP3/caspase-1/IL-1β pathway rather than via IFNs-related pathway.

## Conclusions

Taken together, our data highlighted the potential of inhibition of NLRP3 inflammasome activation as a viable therapeutic approach for the treatment of PM affected by inflammatory disorders where NLRP3 inflammasome is shown to drive the disease. Further investigations are required to elucidate the molecular mechanisms for the role of NLRP3 activation in regulating MHC-I expression and inflammatory process in PM. Further investigations are also needed to address NLRP3 inflammasome as a therapeutic target for PM clinically.

## Supplementary Information

**Additional file 1: Table S1** Primer sequences of used for quantitative RT-PCR.** Table S2** Clinical data of the PM patients and controls.** Fig. S1** No positive staining for IFN-α, IFN-β or IFN-γ was observed in samples from PM patients (n = 3) and controls (n = 3). Scale bars, 50 μm (original magnification, ×400).** Fig. S2** IFNs have weak effects on upregulation of MHC-I expression. IFN-α (80 pg/ml), IFN-β (200 pg/ml), IFN-γ (60 pg/ml), or IL-1β (1 ng/ml) was added into the C2C12 cells for 48 h. The expression of MHC-I in C2C12 cells was analyzed by western.

## Data Availability

The datasets and materials used in the current study are available in the supplementary material.
