# Supplementary material for: NLRP3 inflammasome up-regulates major histocompatibility complex class I expression and promotes inflammatory infiltration in polymyositis
Source: BMC Immunol. 2022 Aug 14;23:39. doi: 10.1186/s12865-022-00515-2 (PMC9375941; doi:10.1186/s12865-022-00515-2)
Supplement: Supplementary file 1 — Additional file 1: Table S1 Primer sequences of used for quantitative RT-PCR. Table S2 Clinical data of the PM patients and controls. Fig. S1 No positive staining for IFN-α, IFN-β or IFN-γ was observed in samples from PM patients (n = 3) and controls (n = 3). Scale bars, 50 μm (original magnification, ×400). Fig. S2 IFNs have weak effects on upregulation of MHC-I expression. IFN-α (80 pg/ml), IFN-β (200 pg/ml), IFN-γ (60 pg/ml), or IL-1β (1 ng/ml) was added into the C2C12 cells for 48 h. The expression of MHC-I in C2C12 cells was analyzed by western. [file 12865_2022_515_MOESM1_ESM.docx]

Supplementary Material

**Additional file 1: Table S1.** **Primer sequences of used for quantitative RT-PCR**

| **Genes** | **Primer sequences (F/R)** |
| --- | --- |
| MHC-I | 5’-TATAAAGTCCACGCAGCCCG-3’  5’-AATACCTCAGCGAGTGTGGG-3’ |
| GAPDH | 5’-ACCCAGAAGACTGTGGATGG-3’  5’-CACATTGGGGGTAGGAACAC-3’ |

**Additional file 1: Table S2.** **Clinical data of the PM patients and controls**

|  | **PM (n = 27)** | **Controls (n = 12)** | ***P*-value** |
| --- | --- | --- | --- |
| **Sex (M/F)** | 9/18 | 5/7 | 0.723 |
| **Age (years)** | 44.4 ± 8.9 | 41.6 ± 10.7 | 0.401 |
| **MYOACT** | 5.3 ± 1.9 | NA | - |
| **pVAS** | 5.8 ± 1.5 | NA | - |
| **MMT-8** | 67.3 ± 6.8 | NA | - |
| **ESR (mm/h)** | 43.2 ± 18.3 | 13.5 ± 5.2 | <0.001 |
| **CRP (mg/L)** | 24.7 ± 5.8 | 15.2 ± 4.5 | <0.001 |
| **CK (U/L)** | 2682.9 ± 1074.2 | 336.5 ± 130.2 | <0.001 |
| **LDH (IU/L)** | 412.5 ± 52.7 | 211.8 ± 78.5 | <0.001 |

PM, polymyositis; MYOACT, myositis disease activity assessment visual analogue scale; NA, not applicable; pVAS, physicians’ global activity assessment on a 10 cm visual analogue scale; MMT-8, manual muscle testing 8; ESR, erythrocyte sedimentation rate; CRP, C-reactive protein; CK, creatine kinase; LDH, lactate dehydrogenase.

**
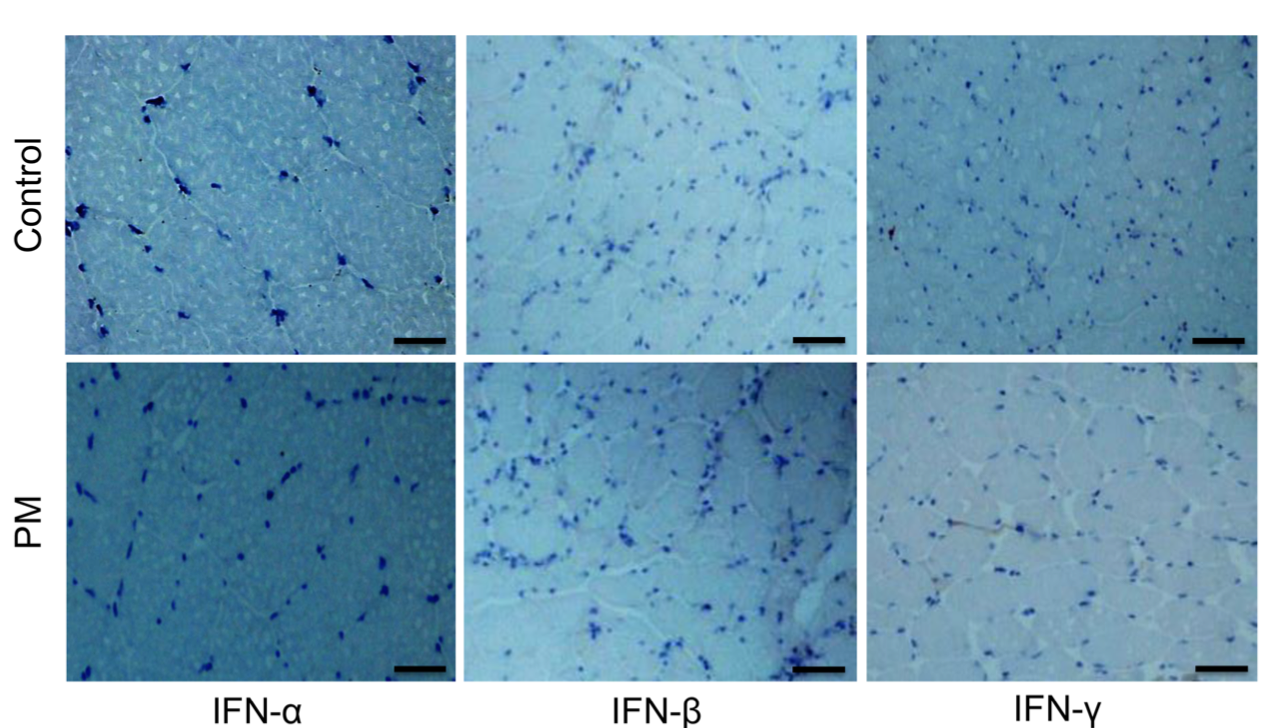
**

**Additional file 1: Fig. S1│**No positive staining for IFN-α, IFN-β or IFN-γ was observed in samples from PM patients (n=3) and controls (n=3). Scale bars, 50 μm (original magnification, ×400).


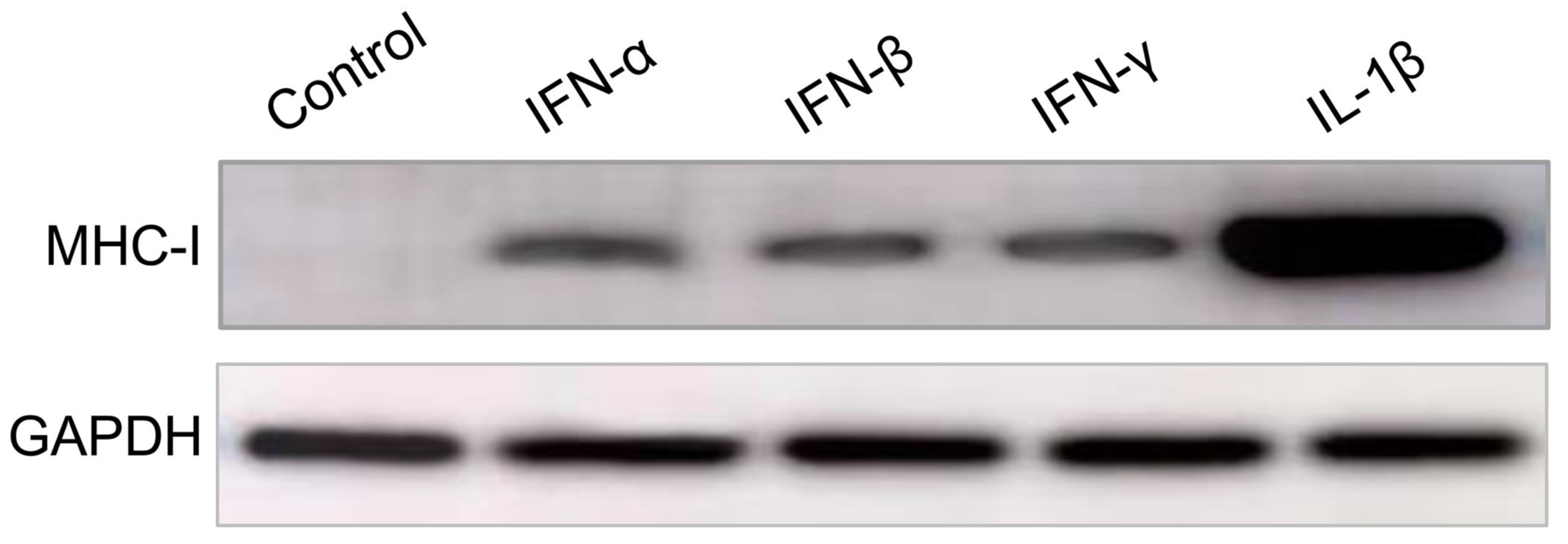


**Additional file 1: Fig. S2│**IFNs have weak effects on upregulation of MHC-I expression. IFN-α (80 pg/ml), IFN-β (200 pg/ml), IFN-γ (60 pg/ml), or IL-1β (1 ng/ml) was added into the C2C12 cells for 48 h. The expression of MHC-I in C2C12 cells was analyzed by western
